# Thermal, mechanical, and moisture absorption properties of egg white protein bioplastics with natural rubber and glycerol

**DOI:** 10.1186/2194-0517-2-12

**Published:** 2013-07-03

**Authors:** Alexander Jones, Mark Ashton Zeller, Suraj Sharma

**Affiliations:** 1 Department of Textiles, Merchandising and Interiors, University of Georgia, Athens, GA 30602 USA; 2 ALGIX, LLC, Athens, GA 30602 USA

**Keywords:** Bioplastics, Albumin, Sustainability, Plasticizers

## Abstract

Petroleum-based plastics have many drawbacks: the large amount of energy required to produce the plastic, the waste generated as a result of plastic production, and the accumulation of waste due to slow degradation rate. It is because of these negative attributes of conventional plastic use that attention is being focused on environmentally friendly plastics from alternative sources. Albumin protein provides one possible source of raw material, with inherent antimicrobial properties that may make it suitable for medical applications. We conducted this study to investigate the various bioplastic properties of the albumin with the use of three plasticizers - water, glycerol, and natural rubber latex. Based on results, 75:25 albumin-water, 75:25 albumin-glycerol, and 80:20 albumin-natural rubber were the best blending ratios for each plasticizer for a subsequent time study to determine water stability, with the 80:20 albumin-natural rubber blend ratio having possessed the best thermal, tensile, and viscoelastic properties overall.

**Electronic supplementary material:**

The online version of this article (doi:10.1186/2194-0517-2-12) contains supplementary material, which is available to authorized users.

## Introduction

Using conventional plastics comes with a multitude of drawbacks: the large amount of energy that is required to produce the plastic, the waste that is a result of plastic production, and the use of materials that do not biodegrade readily. In order to shift the production of plastics towards a more sustainable path, research is being conducted to determine the types of renewable bioplastic resources that could be converted into plastic form. For instance, polylactide biopolymer, one of the few resourceful polymers, is naturally produced on a large scale (Mukerjee 2011). A common theme for various bioplastics that will replace conventional plastics is their tendency to be biodegraded, compared to petroleum-based plastics that are resistant to chemical and biological attacks. According to a review study by Flieger et al. (2003), there are three groups of biodegradable polymers that can be utilized in the production of bioplastics: biopolymers by chemical synthesis, biopolymers through fermentation process by microorganisms, and biopolymers from chemically modified natural products. Under the classification of chemically modified natural products falls the use of protein in producing bioplastics as protein must be modified chemically by the addition of plasticizers and the use of thermal treatments. The main initiative for the use of modified natural products in bioplastics is the continual drive to find more uses for agricultural commodities (Flieger et al. 2003).

It is necessary to determine the thermal and mechanical properties of bioplastics produced from protein as this will help identify the process by which the bioplastic should be made as well as what applications the resulting plastic will be suitable for. In a study by Sharma et al. (2008), they determined that the albumin from chicken egg white denatures at a temperature of 136.5°C ± 3°C. This indicates that in order to produce plastic from the chicken egg white albumin, the material must be molded at 136.5°C ± 3°C to ensure that the protein will be denatured and able to orient and form a bioplastic. When the tensile properties of the protein-based plastics were measured, it was determined that the breaking of hydrophobic interactions and hydrogen bonds of the bioplastics initiated a reversible yield point (Sharma et al. 2008). This reversal of the yield point allows tensile stress to be placed onto the bioplastic multiple times as long as the breaking point is not reached.

In order to determine the potential uses of protein-based bioplastics, thermal and mechanical properties must be examined. Since the protein-based bioplastics require a lower processing temperature and possess tensile properties similar to plastics like high-density polyethylene (HDPE), it is possible to manufacture a bioplastic at a lower production cost (Jerez et al. 2007). However, one potential drawback of using protein in plastics is its hygroscopic properties as it was determined that the water absorption of various bioplastics ranged from 40% to 320% (Jerez et al. 2007). This tendency for bioplastics to absorb water may result in plastic with lower elasticity as the moisture content may alter the elastic modulus of the resulting plastic. Another potential drawback that arises when using protein-based (or any polymer) materials is the lack of knowledge about how the materials will react to bacteria in their environment. In a study by Hook et al. (2012), they determined that certain polymers were actually better at preventing microbial growth than either the silicone or silver hydrogel coatings that are commonly applied to medical devices before being implanted into humans. One other potential drawback with bioplastics is permanent deformation when stress is applied (Widiastuti et al. 2013). Although researchers have utilized composites to decrease the amount of deformation and creep, research must still be conducted in order to examine both the positive and negative properties of protein bioplastics in order to determine if they are suitable for certain applications (Dorigato and Pegoretti 2012).

Certain constituents of albumin pose a potential advantage of possessing inherent antibacterial properties allowing for potential pharmaceutical and medicinal uses. Albumin possesses this property most notably from its lysozyme enzyme constituent, utilizing a lysis reaction in which it will break down the peptidoglycan barrier of bacteria consisting of the glycosidic (1–4) β-linkage between the *N*-acetylglucosamine and the *N*-acetylmuramic acid (Baron and Rehault 2007). It is also possible to improve the antimicrobial properties of the lysozyme enzyme through the use of chemical modifications. There are various preservatives such as nisin and sodium lactate as well as substances such as ethylenediaminetetraacetic acid, butylparaben, and trisodium phosphate that can be added to the lysozyme to enhance its properties (Cegielska-Radziejewska et al. 2010). The utilization of albumin in medical plastic production would not be an extension on what chicken albumin is being used for today. It is because of inherent antibacterial enzymes and improvement through chemical modification that albumin is already used in various medical applications, such as circulatory support, drug delivery, and the removal of toxins from the body (Peters 1996). Another area in which albumin bioplastics or its thermoplastic blends (with petroleum-based polymers/biopolymers/biodegradable polymers) could be utilized is in drug elution as well as orthopedic implants and sutures as it would serve as a material that could release a low dose of medicine over a period of time in the body while limiting the risk of infection (Zilberman and Elsner 2008). Our objectives when conducting this study were to modify the properties of albumin plastics through the use of glycerol and natural rubber latex and to evaluate the physical performance of these plastics as time progressed.

## Methods

### Materials

The albumin (purity ≥99%) utilized in the production of bioplastics was obtained from Sigma-Aldrich Corporation (St. Louis, MO, USA). The plasticizers used to form the bioplastics were obtained through various sources: deionized water was obtained through filtering water in the lab, glycerol was obtained from Sigma-Aldrich with a purity ≥99%, and natural rubber latex (70% solid, 30% water mixture with a pH of 10.8) was obtained from the Chemionics Corporation (Tallmadge, OH, USA).

### Preparation of compression-molded samples

Molding of the albumin-based bioplastic blends were performed on a 24-ton bench-top press (Carver Model 3850, Wabash, IN, USA) with electrically heated and water-cooled platens. The stainless steel molds were custom made to form either dog bone-shaped bioplastics for mechanical analysis or two small rectangular flex bars for various property analyses. Data presented in this study was generated from compression-molded samples using a 5-min cook time at 136.5°C followed by a 10-min cooling period, under a pressure of at least 40 MPa as a certain minimum amount of pressure was required in order to mold plastic (Sue et al. 1997). The bioplastic blends were prepared in small batches of ≤6 g and then poured into the molds at a constant weight, with dynamic mechanical analysis (DMA) flex bars made of 2 g and dog bones made of 6 g of albumin powder. After the samples were cooled for 10 min under pressure, the pressure was released and the samples were removed. The samples were then placed in a conditioning chamber for at least 24 h, unless otherwise noted. The conditioning chamber was set to 21.1°C and 65% relative humidity.

### Weight change and moisture content analysis

The bioplastic samples were placed in conditioning chamber settings to determine moisture content over time - initial, 1, 2, 3, 4, 5, 6, 24, 48, 72, and 96 h after molding. In order to ensure accurate measurements, four DMA flex bars were prepared and analyzed during this process. Moisture content of plastics were analyzed by cryocrushing bioplastics with liquid nitrogen for each blend type (*n* = 4) and heated at 80°C for 1 h, with 10 min of cooling afterwards. The equation used to determine the moisture content was as follows:

$$\mathrm{MC}=\left[\left(W_{0}-W_{0d}\right)/W_{0}\right]\times 100,$$

where *W*_0_ = initial weight of specimen and *W*_0*d*_ = weight of specimen after drying.

### Dynamic mechanical analysis

After conditioning, DMA flex bars were analyzed for their viscoelastic properties through the use of dynamic mechanical analysis (Menard 1999) using a DMA 8000 dynamic mechanical analyzer from PerkinElmer (Branford, CT, USA) starting at a temperature of 25°C and ending at a temperature of 160°C, with a temperature ramp of 2°C min^−1^. The settings of the analyzer were set to dimensions of 9 × 2.5 × 12.5 mm^3^ using a dual cantilever setup at a frequency of 1 Hz with a displacement of 0.05 mm. Each sample type was analyzed in duplicate (*n* = 2) to ensure accuracy. The DMA flex bars were also tested in intervals of immediate, 24 h, and 5 days after molding in order to determine the viscoelastic properties over time.

### Thermal analysis

Thermal gravimetric analysis (TGA) was performed using a Mettler Toledo TGA/SDTA851e (Columbus, OH, USA), and differential scanning calorimetry (DSC) was performed using a Mettler Toledo DSC821e. TGA was performed from 25°C to 800°C under N_2_ atmosphere with a heating rate of 10°C min^−1^. DSC was performed from −50°C to −250°C under N_2_ atmosphere with a heating rate of 20°C min^−1^. All samples (*n* = 2) were prepared with weights between 2.0 and 4.0 mg as the samples were cut from DMA flex bars for each blend. TGA and DSC tests were conducted in intervals of immediate, 24 h, and 5 days after molding.

### Scanning electron microscopy

Albumin scanning electron microscopy (SEM) samples (*n* = 2 for each plastic type) were prepared from cryogenic DMA flex bar fracture surfaces after being placed in a conditioning chamber setting for at least 24 h. DMA flex bars were submerged in liquid nitrogen for 20 s; after that, they were immediately broken. The samples were mounted, then sputter-coated for 60 s with an Au/Pt mix. SEM images were recorded on a Zeiss 1450EP (Carl Zeiss AG, Oberkochen, Germany) variable pressure scanning electron microscope. Coated samples were analyzed at × 20, ×100, and × 500 for each blend type.

### Mechanical properties

The mechanical properties of the conditioned albumin bioplastics were measured using the Instron testing system (Model 3343, Instron Corporation, Norwood, MA, USA) interfaced with the Blue Hill software. The test was performed according to the standard test method for tensile properties of plastics (ASTM D 638–10, type I) with a 5-mm min^−1^ crosshead speed, a static load cell of 1,000 N, and a gauge length of 4 cm. Samples were run in quintuplicate (*n* = 5) for each blend type in order to ensure accurate measurement.

### Statistical methods

Statistical analyses of data were generated for moisture content analysis and mechanical property analysis through the use of power analysis. For each plastic type tested, statistical values based on the mean and standard deviation were generated, with *p* values (0.05 or less) compared to plastic types based on properties being tested generated from Student’s *t* test distribution. For moisture content analysis, correlation analysis was also conducted (1 = perfect positive correlation, 0 = no correlation, −1 = perfect negative correlation).

## Results and discussion

### Initial material analysis

#### Thermal properties of albumin

**Figure 1:**
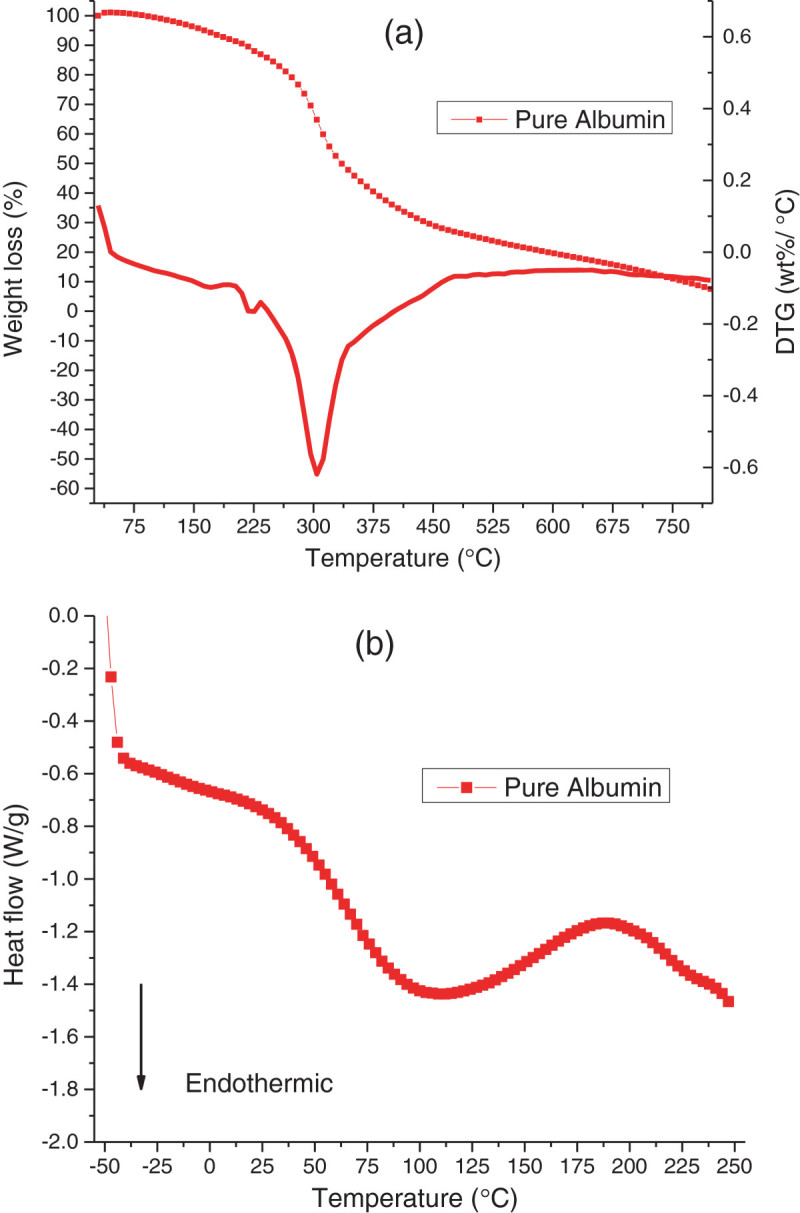
**Thermographs of pure albumin powder. (a)** TGA and **(b)** DSC.

An initial degradation peak was shown between 220°C and 230°C, with a much larger peak starting from 245 to 250°C, and 93% of the albumin powder degraded by the end of the TGA run (Figure 1). These results were similar to the results obtained in the work conducted by Sharma and Luzinov (2012). For DSC data, the endothermic dip began at 75°C with a broad peak between 120°C and 125°C. This indicated that the material had fully passed its transition phase - denaturation. An endothermic decomposition or pyrolysis peak occurred at 250°C, which exhibited the onset of degradation. Therefore, the albumin-based bioplastics were molded at 136.5°C as this was the safe temperature of processing albumin into the plastics with as little degradation occurring as possible. Based on the albumin being fully denatured between 120°C and 125°C without degradation, it was determined that the plastics were to be molded higher than this temperature but below temperatures where degradation occurs (Figure 1).

#### Dynamic mechanical analysis

**Figure 2:**
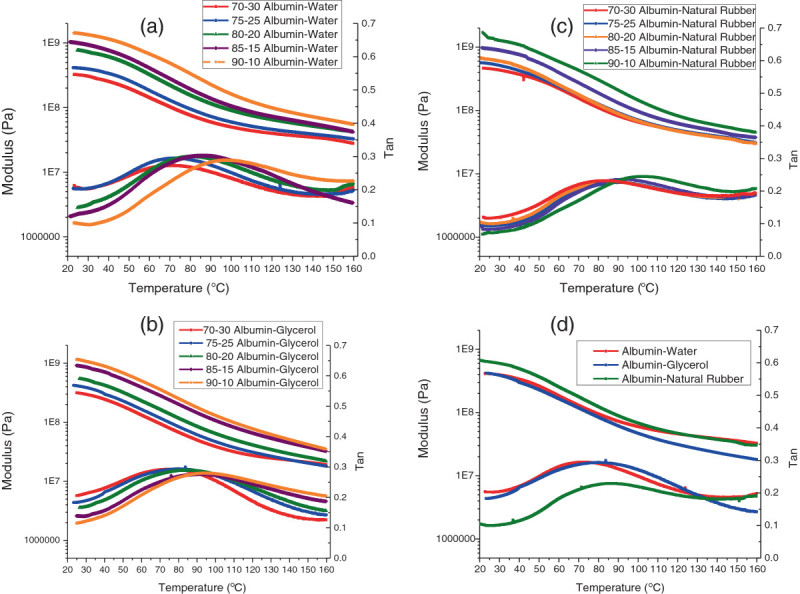
**Dynamic mechanical analysis of initial albumin plastics. (a)** Albumin-water, **(b)** albumin-glycerol, **(c)** albumin-natural rubber, and **(d)** optimum blends of each plastic.

In plastics with water as a plasticizer, we found that as the amount of water was increased, the initial modulus of the resulting plastics decreased, with the tanδ peak occurring at 70°C (Figure 2a). This was consistent with the research conducted by González-Gutiérrez et al. (2011). The increased water content caused an increase in the initial tanδ values as well as caused the tanδ peaks to shift to the left (or lowered glass transition temperature) and occurred at lower temperatures, which indicated increased viscous heat dissipation. The shifted curves indicated that the 75:25 albumin-water formulation was the most desirable of the blends examined as this formulation possessed the mix of a modulus that was comparable to the other water plasticized samples (and higher than the 70:30 albumin-water formulation), while possessing an elasticity (tanδ) that was much higher than the other formulations (and equal to the 70:30 albumin-water formulation), as shown in Figure 2a. The same trends occurred in the albumin plastics that had glycerol as a plasticizer - the higher percentage led to the higher initial tanδ and lower modulus as well as the shifting of the tanδ peaks to the left (Figure 2b). However, at lower content of both water and glycerol, the bioplastics showed anti-plasticization and plasticization phenomena (Galdeano et al. 2009). Based on the results, we determined that the 75:25 albumin-glycerol ratio was the composition with the highest overall tanδ peak as well as moderate modulus values (Figure 2b).

For the albumin plastics with natural rubber latex as the plasticizer, we observed the same trends, although there was very little difference in the initial tanδ values (Figure 2c). The 80:20 albumin-rubber formulation possessed the optimal mix of high initial modulus and tanδ as its tanδ values were comparable to the 70:30 and 75:25 albumin-rubber ratios. However, the 80:20 albumin-rubber bioplastics possessed a higher initial modulus while having a tanδ peak at a lower temperature than the bioplastics that contained lower weights of rubber (Figure 2c). When we compared the plastics based on the types of plasticizer used, we found that the initial modulus was similar for all three plasticizers, but the natural rubber-based bioplastics exhibited the lowest initial tanδ values, whereas other plasticizers (water and glycerol) showed highest viscous heat dissipation (Pommet et al. 2005). With this analysis completed, it was determined that the optimum blends for albumin plastic production were 75:25 albumin-water, 75:25 albumin-glycerol, and 80:20 albumin-rubber (Figure 2d).

### Time study

#### Bioplastic moisture content analysis

**Figure 3:**
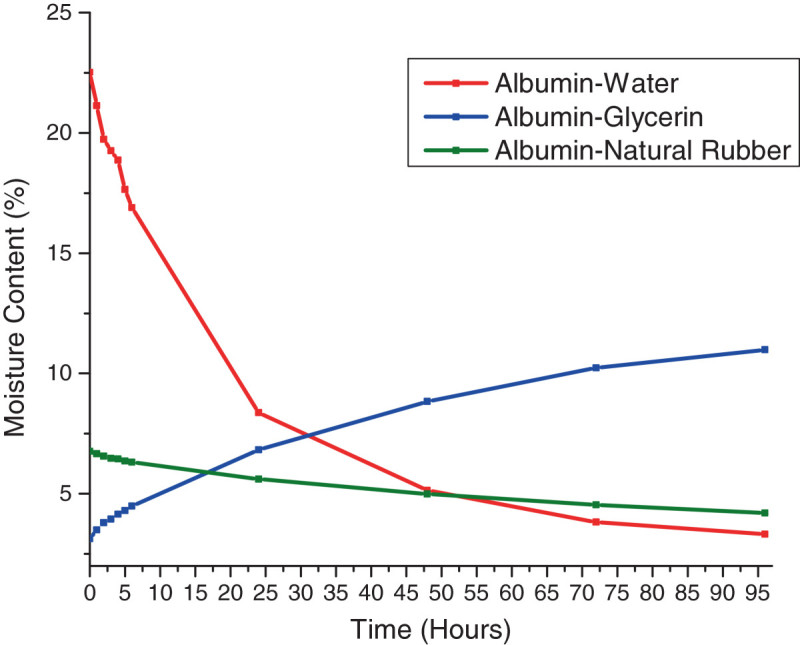
**Moisture content of albumin plastics over time.**

The 75:25 albumin-water bioplastics demonstrated a large decrease in moisture content within 48 h of molding, losing an average between 15% and 17% of its initial moisture content (Figure 3). However, after the initial loss of moisture content, the plastics maintained a stable moisture content after 96 h of molding. This loss in moisture content was due to the loss of water from the bioplastic as time progressed, producing a stiffer and more brittle plastic (Van Soest and Knooren 1997). While the water-based bioplastics lost moisture content, the 75:25 albumin-glycerol formulation steadily increased in moisture content after molding, reaching 10% in moisture content 96 h after molding as the time progression correlated (0.978) with moisture content growth. The gain in moisture content was due to a combination of glycerol leaching from the bioplastics and the plastic absorbing ambient moisture. This could render this plastic unsuitable for most applications as the resulting water absorption would alter the properties of the plastic, reducing its tensile strength. While the water- and glycerol-based plastics underwent a comparatively large change in moisture content during the study (*p* = 0.002), the 80:20 albumin-rubber bioplastics were more stable in terms of moisture content, maintaining a moisture content between 5% and 7% on average throughout the study, as it was significantly equivalent to the albumin-glycerol values (*p* = 0.472). This moisture content stability was due to natural rubber being stable and nonreactive in the environment, with the only moisture loss due to the loss of water that was in the natural rubber latex molding base (Carvalho et al. 2003).

Based on our findings in the study, it was determined that the natural rubber provided the most stability in terms of moisture content as the other plasticizers either lost moisture over time (water-based bioplastic) or gained moisture (glycerol-based bioplastic) (Figure 3).

#### Bioplastic dynamic mechanical analysis

**Figure 4:**
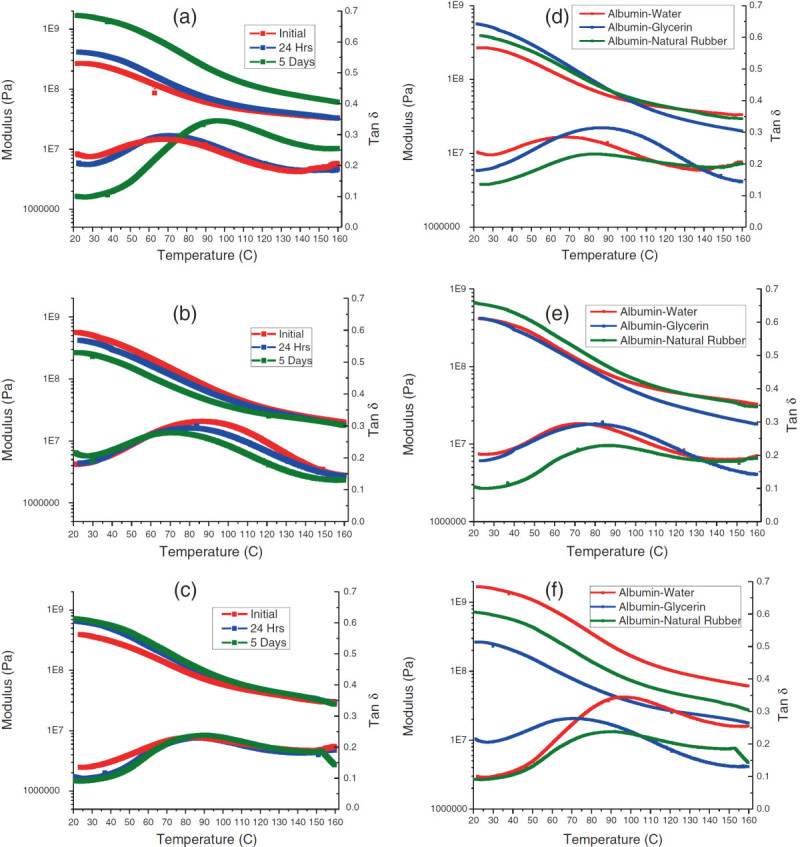
**Dynamic mechanical analysis of time study on albumin plastics. (a)** Albumin-water plastics, **(b)** albumin-glycerol plastics, **(c)** albumin-natural rubber plastics, **(d)** initial plastics, **(e)** 24-h plastics, and **(f)** 5-day plastics.

The 75:25 albumin-water plastics showed the most significant amount of change: the modulus drastically increased after 5 days of conditioning (initial = 2.5E8 Pa, 5 days = 1.8E9 Pa) with lower initial tanδ values (initial = 0.23, 5 days = 0.09), and the tanδ peak shifted to the right (Figure 4;Van Soest and Knooren 1997). These characteristics pointed to the unbound water being released over time when it was placed in ambient conditions, which reduced the ability of water to plasticize, as shown in past studies (Verbeek and van den Berg 2010). The change in properties of these plastics over time was most likely due to the amount of moisture loss that occurred over time, which resulted in a stiff plastic. This drastic change in the properties of albumin-water plastics over time pointed to a lack of usability in the material as materials must maintain consistent properties for more than a short period of time (Figure 4a). The 75:25 albumin-glycerol plastics demonstrated almost completely opposite results, though the amount of change over time was not as drastic (Figure 4b). Conditioning for the glycerol-based plastics led to the lowering of initial modulus (initial = 5.4E8 Pa, 5 days = 2.6E8 Pa) and a slight increase in tanδ (initial = 0.17, 5 days = 0.21) as well as the general lowering and shifting to the left of the tanδ peak. These changes in viscoelastic properties were most likely due to the gradual leaching of glycerol from the plastic, with ambient moisture taken in to replace it, weakening the hydrogen bonds within the plastic in the process (Lodha and Netravali 2005). When it came to the 80:20 albumin-rubber plastics, there was very little change that occurred in the plastic after it was allowed to condition for 24 h as the tanδ and modulus values were essentially identical after the plastics were given time to condition (Figure 4c). This lack of change in properties may have been due to natural rubber lacking the ability to react to the environment; if given enough time, it will remain bonded to the albumin and maintain its basic properties (Carvalho et al. 2003).

When the plasticizers are compared, the 75:25 albumin-water plastics gradually became the most brittle over time as their modulus increased and tanδ decreased. The 75:25 albumin-glycerol plastics underwent the exact opposite in properties as they initially started as the most brittle of the plastics but at the end of the experiment became the most ductile due to its loss of modulus and maintaining its initial tanδ value. As for the 80:20 albumin-natural rubber plastics, they maintained the most consistent among the plastics as after the decrease of tanδ and increase of modulus after 24 h, their viscoelastic properties normalized (Figure 4d,e,f).

#### Bioplastic thermal analysis

**Figure 5:**
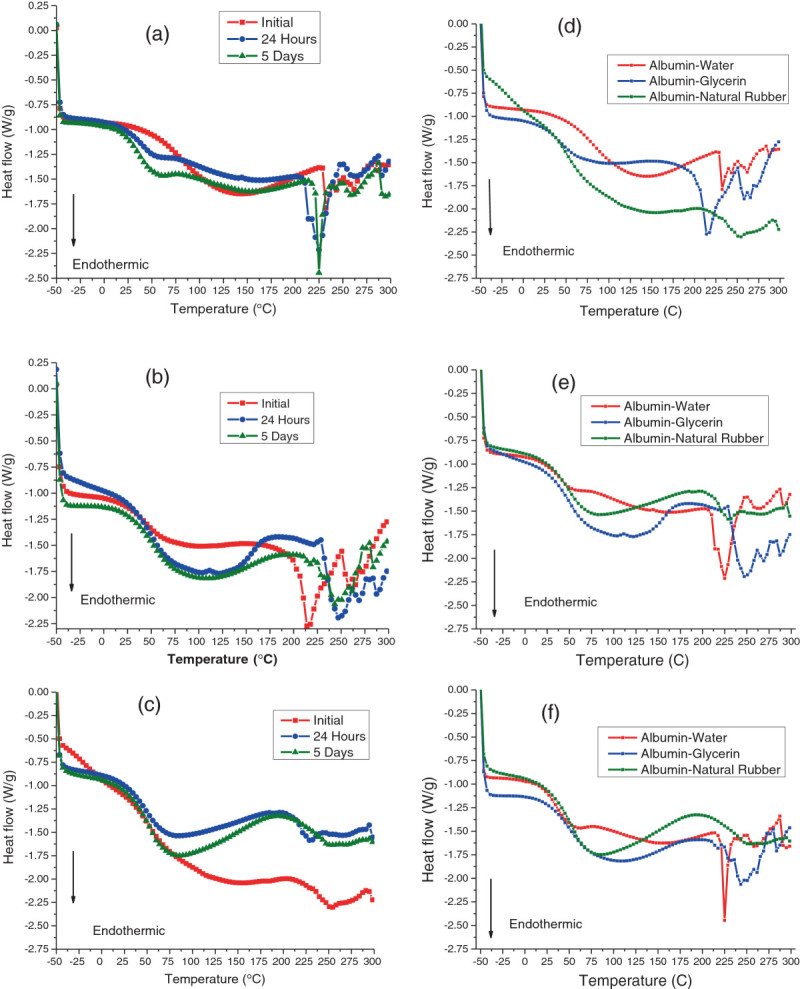
**Differential scanning calorimetry of time study on albumin plastics. (a)** albumin-water plastics, **(b)** albumin-glycerol plastics, **(c)** albumin-natural rubber plastics, **(d)** initial plastics, **(e)** plastics after 24 h, and **(f)** plastics after 5 days.

In the 75:25 albumin-water plastics, the glass transition temperature range of 40°C to 60°C was more evident in the 24-h and 5-day samples (Figure 5a), while for all three samples, an endothermic peak was seen around 225°C. This peak in the albumin-water bioplastics could have been attributed to the degradation or decomposition of protein polymers. For the 75:25 albumin-glycerol plastics, the glass transition phase of 50°C to 110°C was also very noticeable, with a small dip beginning at180°C (due to glycerol) and a larger endothermic peak between 215°C and 220°C for bioplastics that had been molded on the same day (Figure 5b). The larger endothermic peaks were at 250°C for the 24-h and 5-day samples. This shift in protein decomposition to higher temperature could have been attributed to the absorption of moisture and reorganized polymer chains due to the displacement of unbound glycerol molecules (Chen et al. 2005). As for the 80:20 albumin-rubber samples, a much clearer glass transition of 40°C to 80°C occurred with the 24-h and 5-day samples in comparison with the initial sample, with all three samples beginning to have an endothermic decomposition peak at 225°C (Figure 5c).

When the plastics were compared with each other, it was found that the natural rubber-based bioplastics underwent a much more noticeable endothermic enthalpy change initially, but after conditioning, it recovered to normal glass transition phase, similar to that in other plasticizers used (Figure 5d,e,f).

**Figure 6:**
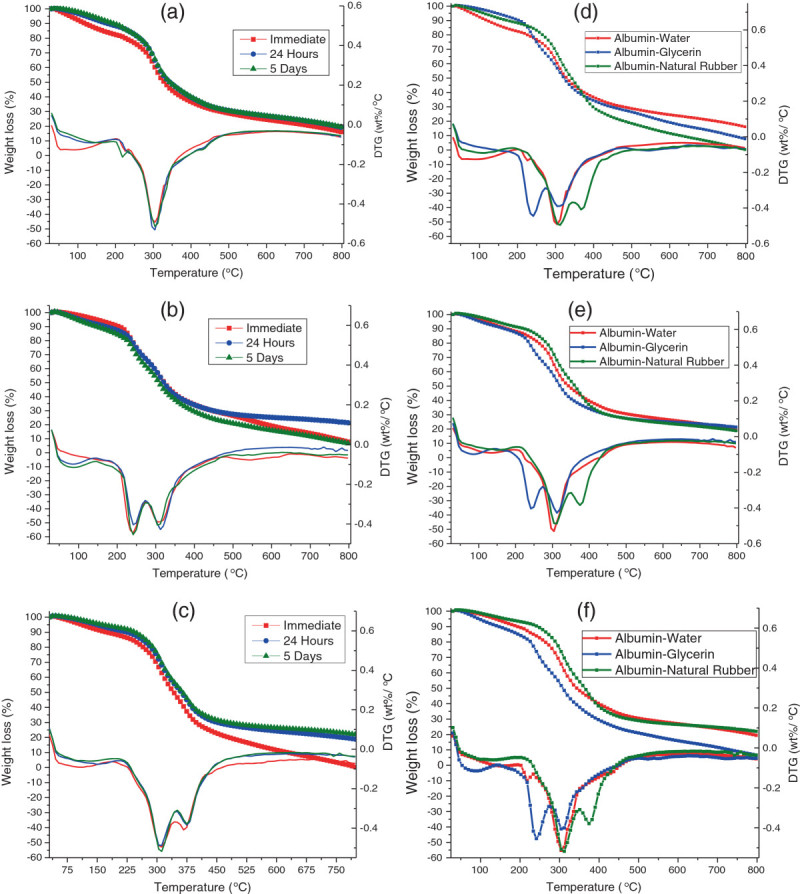
**Thermogravimetric analysis of time study on albumin plastics. (a)** Albumin-water plastics, **(b)** albumin-glycerol plastics, **(c)** albumin-natural rubber plastics, **(d)** initial plastics, **(e)** plastics after 24 h, and **(f)** plastics after 5 days.

In terms of the thermogravimetric analysis, we found that the amount of time after molding did not have an effect on the amount of mass loss at higher temperatures as all of the curves were similar depending on the type of plasticizer used. The 75:25 albumin-water plastics possessed one degradation peak at 300°C, where the protein within the plastic begins to degrade, while a small drop at the beginning of each curve was due to moisture loss (Figure 6a). This moisture loss was more evident in the initial plastic, as this sample had the highest amount of moisture, resulting in a mass drop between 50°C and 100°C. For the 75:25 albumin-glycerol plastics, there was one bimodal degradation peak for all of the samples (Figure 6b). The first peak began at 225°C, which was most likely due to glycerol degradation (flashpoint of glycerol is around 180°C, with mass loss occurring in nitrogen gas environments at 199°C (Castelló et al. 2009)) within the bioplastic; the peak was right-shifted, most likely due to stabilization in the albumin matrix. The second peak between 300°C and 325°C was most likely due to the albumin protein itself degrading. The 80:20 albumin-rubber samples also possessed a bimodal degradation peak, although we found that the peaks were in different temperature ranges compared to one in glycerol-plasticized bioplastics (Figure 6c). For instance, the first peak seen at 300°C was the initial degradation of the protein in the plastic. However, the second peak seen at a higher temperature at 375°C was most likely due to natural rubber latex degradation, with the rubber degrading between 350°C and 360°C (Mathew et al. 2001). The initial sample of natural rubber latex sample also degraded at a higher rate and a slightly lower temperature due to the water contained in the natural rubber latex molding base still being present in the plastic.

When all three plasticizers are compared, we found that as time progressed, the albumin-glycerol plastics possessed the lowest onset degradation temperature, which may have been due to the increase of moisture in the plastic leading to destructurization (Figure 6d,e,f). The albumin-water and albumin-natural rubber plastics showed higher onset degradation temperatures. This was most likely due to the natural latex making cross-links with plastic matrix and the water-based plastic becoming stiffer over conditioning time due to restructured polymer chains. Once the degradation of the plasticizer had occurred, however, it was found that the water and natural rubber plastics would reach about the same rate of mass loss, with the glycerol plastics losing almost all of their mass over the time of the experiment.

Overall, based on the DSC data, it was found that after conditioning for 24 h, the plastics would maintain consistent values. As for the TGA data, it could have been determined that the albumin-natural latex plastics had the highest onset degradation temperatures, while the 75:25 albumin-glycerol plastics had the lowest temperature for degradation onset.

#### Tensile properties of bioplastics

**Figure 7:**
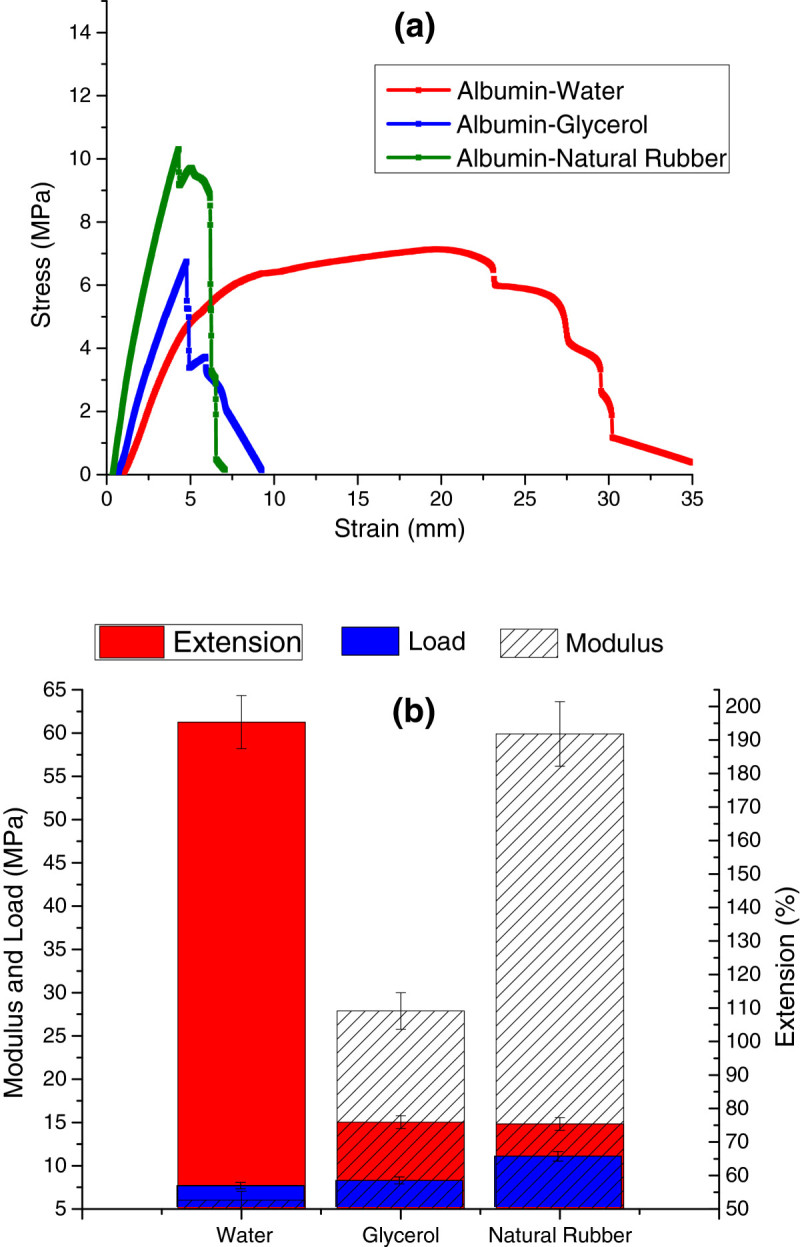
**Tensile properties of time study on albumin plastics after 24 h of conditioning. (a)** Stress–strain curve. **(b)** Modulus, load, and extension chart.

In terms of the amount of extension that occurred before breaking, the 75:25 albumin-water plastics possessed by far the highest amount of extension, extending nearly 200% on average before a ductile break; the other plastics extended around only 75% before breaking (Figure 7) as the glycerol and natural rubber plastics were statistically undistinguishable (*p* = 0.943). One possible reason why water was able to facilitate a higher extension (but not load bearing) could have been due to the bonding that would occur within the structure as plasticization occurred, which had been found in previous research that had been conducted (Pommet et al. 2005;Verbeek and van den Berg 2010). As for the load that was required to break the bioplastics and the modulus of the bioplastic itself, we found that the 80:20 albumin-rubber plastics required a much higher load to break the samples (around 12 MPa) and inherently had a much higher modulus near 60 MPa. For the 75:25 albumin-water and 75:25 albumin-glycerol plastics, the maximum loads that we observed were undistinguishable from each other as a *p* value of 0.757 illustrates. This ability for the albumin-rubber plastics to undergo a high load before plastic deformation may have been due to the natural rubber providing a more load-bearing material in the structure of the plastic, counteracting any potential losses due to long-range plasticization prevention. When the rubber serves as a load-bearing constituent of the plastic, it was possible for the plastic to undergo a higher amount of stress before breaking (Carvalho et al. 2003). Comparing each of the plastic types overall, it was evident that the ductile 75:25 albumin-water and the 80:20 albumin-rubber plastics were the best types of bioplastic to use, depending on the intended use, as the water-based samples allowed large amounts of extension before breaking, while the rubber-based samples were stiff and required the highest amount of load needed to break the samples. As for the brittle 75:25 albumin-glycerol plastics, there was very little benefit in terms of tensile properties as it possessed neither the extension nor the strength that the other plastics possess. The weak tensile properties of the albumin-glycerol plastics could have been explained through disordered conformations as the relatively large chemical structure of glycerol prevented any long-range plasticization to occur (Aman Ullah et al. 2011). When long-range plasticization was prevented, there was a limit on how much force a plastic could have undergone; as when the short polymer chains were broken under stress, it resulted in a violent break at a lower stress than a long chain polymer.

When we examined the tensile properties obtained through this study, we found that the results were similar to the results obtained in the study by Jerez et al. (2007). In their study, they determined that the lower processing temperature of the albumin bioplastic molding process resulted in modulus values that were similar to those of polymers, such as low-density polyethylene (LDPE) and HDPE (Jerez et al. 2007). This discovery made it possible for the use of albumin plastics in place of LDPE and HDPE in certain applications as the reduction of protein as a total percentage of the blend and the lower processing temperature would lower the costs of plastic production.

Based on the results, we determined that the 80:20 albumin-natural rubber plastics were able to undergo the greatest amount of stress, while the 75:25 albumin-water plastics were able to undergo the greatest amount of strain before breaking (Figure 7).

#### Scanning electron microscopy images of bioplastics

**Figure 8:**
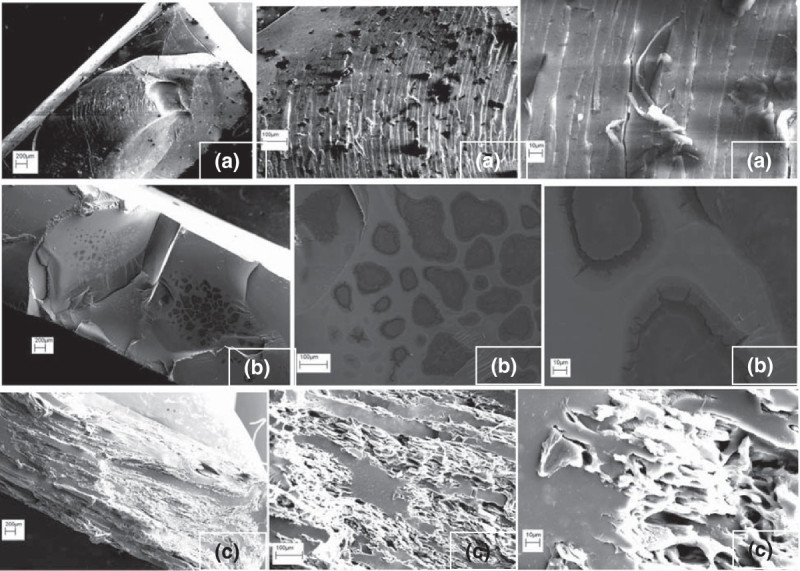
**Scanning electron Microscopy images of albumin bioplastics. (a)** Albumin-water, **(b)** albumin-glycerol, and **(c)** albumin-natural rubber bioplastics. Magnification × 20, ×100, and × 500.

When the 75:25 albumin-water plastics were analyzed through scanning electron microscopy, the pictures illustrated that when the plastic was broken, a scratched and pitted surface was the result, pointing to the fact that the break was not clean and that mixture of the albumin and the plasticizer was fairly homogenous (Figure 8a). This scratching indicated the high level of toughness needed to break the sample, which could also have been seen in the high extension of the albumin-water bioplastics (Figure 7). For the 75:25 albumin-glycerol bioplastics, the spotted surface of the broken plastic was the aspect of highest interest as these images could have been the evidence of glycerol leaching from the plastic on a much smaller scale, with the moisture being removed from the sample when the SEM chamber was sealed under high vacuum (Figure 8b). As the glycerol slowly leached into the environment, pores would form inside the plastic, with moisture from the environment causing the pores to absorb water. When the SEM chamber was sealed and moisture was vacuumed from the testing chamber, cracks formed in the plastic because the moisture was being removed from the plastic. With the 80:20 albumin-rubber bioplastics, what was most evident with these pictures was the lack of homogenous mixture of the albumin and the rubber as there were pockets of albumin and pockets of rubber throughout the whole plastic sample (Figure 8c). The jagged surface of the plastic also illustrated the amount of force that was required to break the plastic as the break would have been sudden and drastic (Carvalho et al. 2003). This abrupt breaking point was also illustrated in the tensile strength and modulus results (Figure 7); the latex was able to hold the plastic together until giving way under a high load. Based on the results of this analysis, we determined that it was glycerol leaching in the 75:25 albumin-glycerol plastics that altered the properties of the plastic, while multiple phases of material in the 80:20 albumin-natural rubber plastics could have been observed.

## Conclusions

When comparing the amounts of plasticizer used in molding bioplastics from albumin, we found that the blending ratios that had the best combination of initial modulus and elasticity were the 75:25 albumin-water, 75:25 albumin-glycerol, and 80:20 albumin-natural Rubber. Of these blends, we found that the 80:20 albumin-natural rubber bioplastic provided the best thermal, viscoelastic, and tensile properties, while properties of the 75:25 albumin-water and 75:25 albumin-glycerol plastics were not comparable due to moisture loss in the water-based bioplastics over time, while glycerol leaching occurred in the glycerol-based bioplastics as time passed. There are multiple avenues of interest that should be examined in the light of the knowledge gained in this study. In order to determine whether the albumin-natural rubber (as well as albumin-water for short-term uses) plastics would be suitable for medical applications or not, further studies are needed to ensure that the plastics will inhibit bacterial growth in order to prevent post-operation infection as well as potentially aid in the application of drugs through elution. Another area of interest requiring further research is the continued modification of the blending by altering the percentages of each component used as well as adding different materials into the blends in order to reach the optimum properties for other applications. As for the aspect of moisture content, it would be beneficial to examine the possible uses of other materials that would limit the amount of moisture content change that would occur with albumin plastics as it has been demonstrated that this area has a significant effect on the overall properties of the plastic.

## Authors’ original submitted files for images

Below are the links to the authors’ original submitted files for images. Authors’ original file for figure 1 Authors’ original file for figure 2 Authors’ original file for figure 3 Authors’ original file for figure 4 Authors’ original file for figure 5 Authors’ original file for figure 6 Authors’ original file for figure 7 Authors’ original file for figure 8
