# Supplementary figures and images for: Thermal, mechanical, and moisture absorption properties of egg white protein bioplastics with natural rubber and glycerol
Source: Prog Biomater. 2013 Jul 3;2:12. doi: 10.1186/2194-0517-2-12 (PMC5151117; doi:10.1186/2194-0517-2-12)

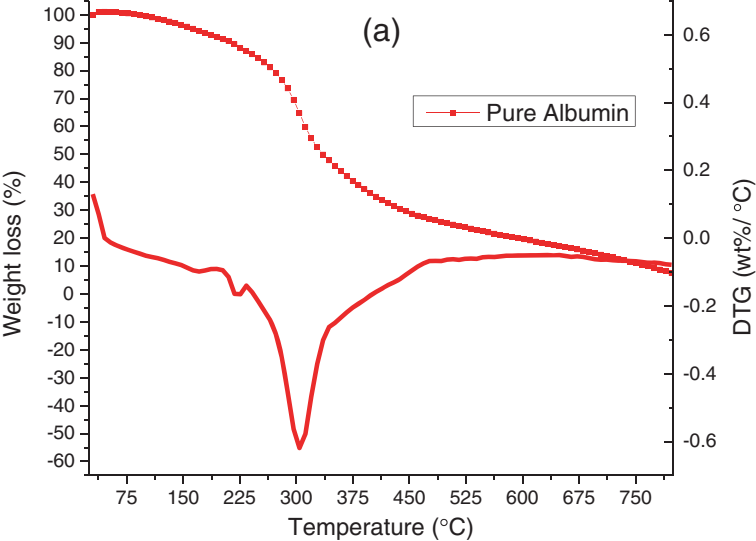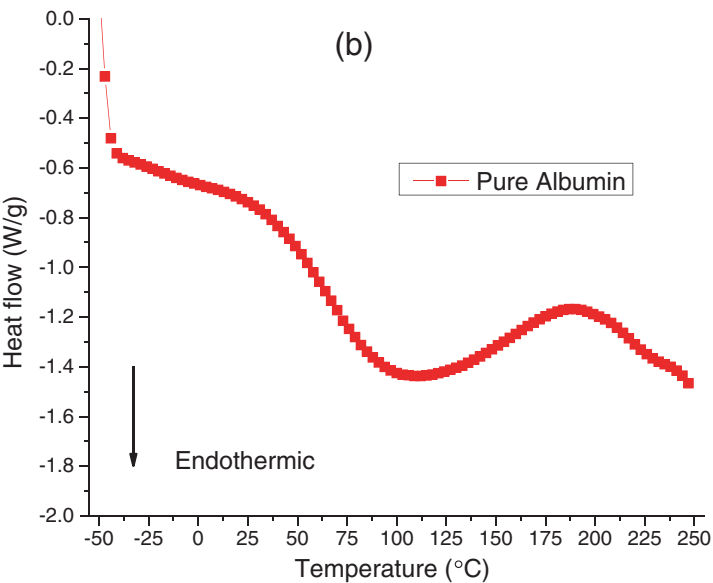

Supplement: Supplementary file 1 — Authors’ original file for figure 1 [file 40204_2013_16_MOESM1_ESM.pdf]

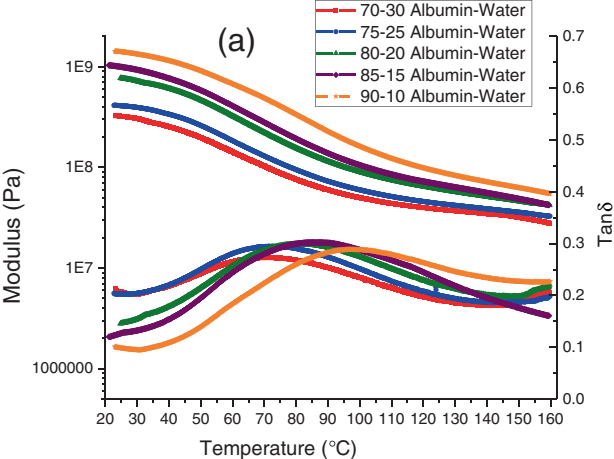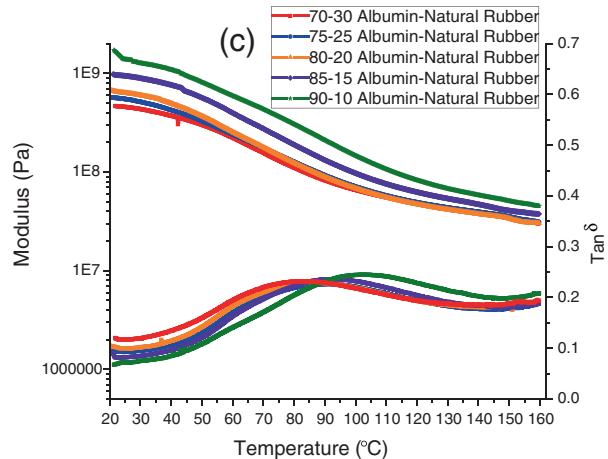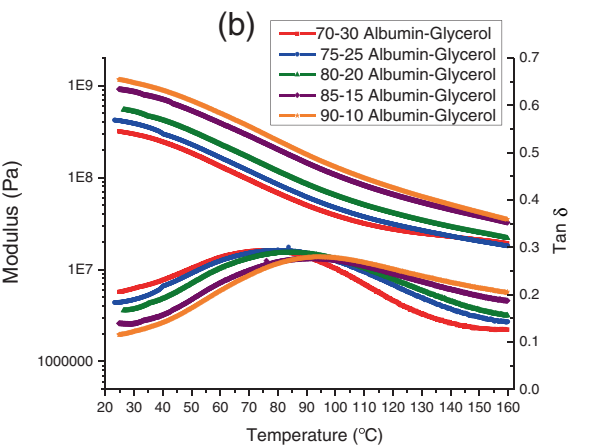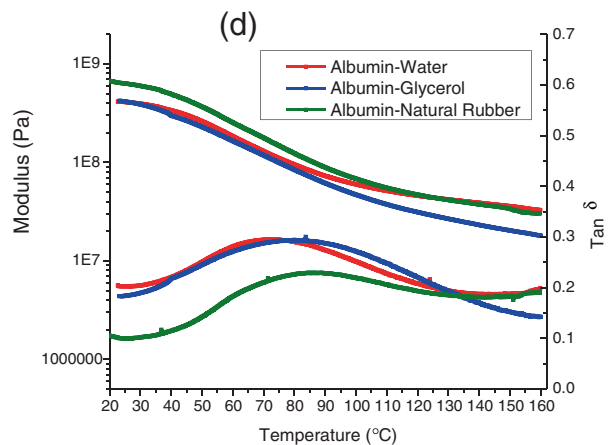

Supplement: Supplementary file 2 — Authors’ original file for figure 2 [file 40204_2013_16_MOESM2_ESM.pdf]

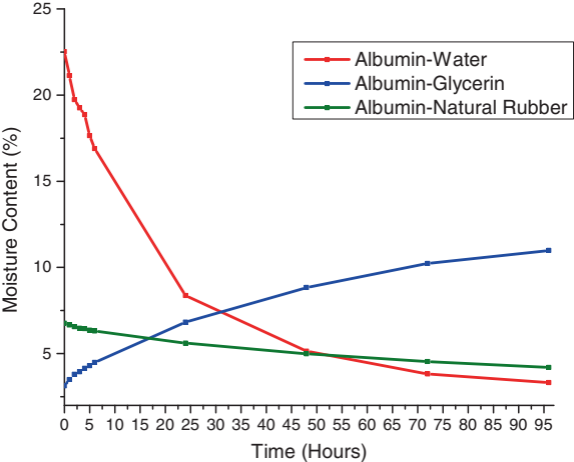

Supplement: Supplementary file 3 — Authors’ original file for figure 3 [file 40204_2013_16_MOESM3_ESM.pdf]

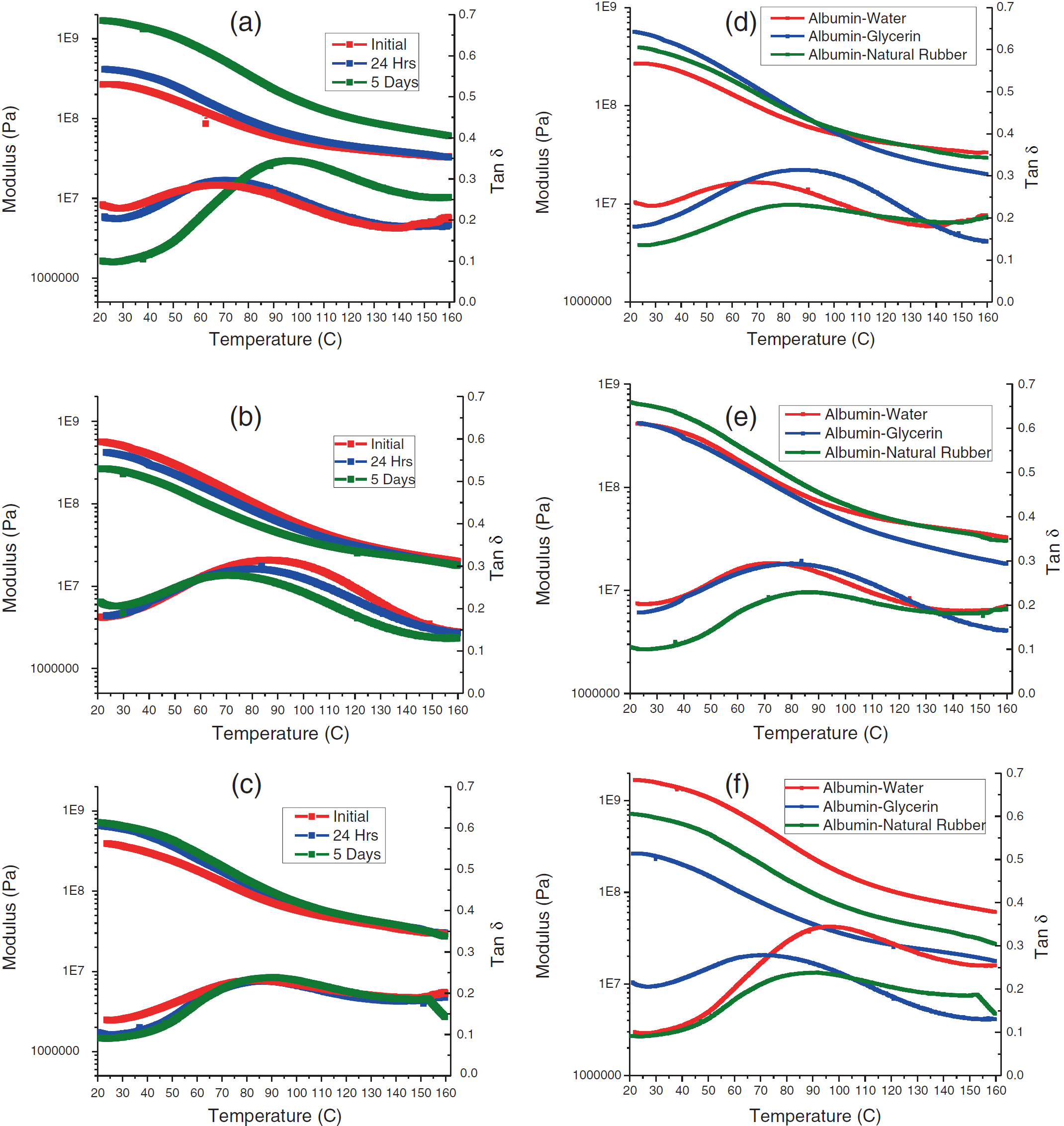

Supplement: Supplementary file 4 — Authors’ original file for figure 4 [file 40204_2013_16_MOESM4_ESM.png]

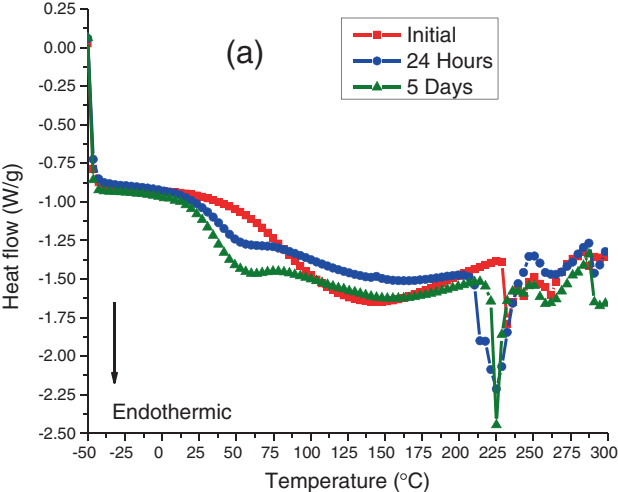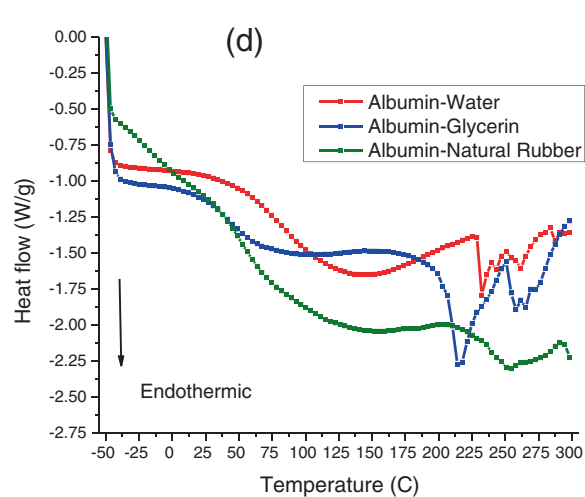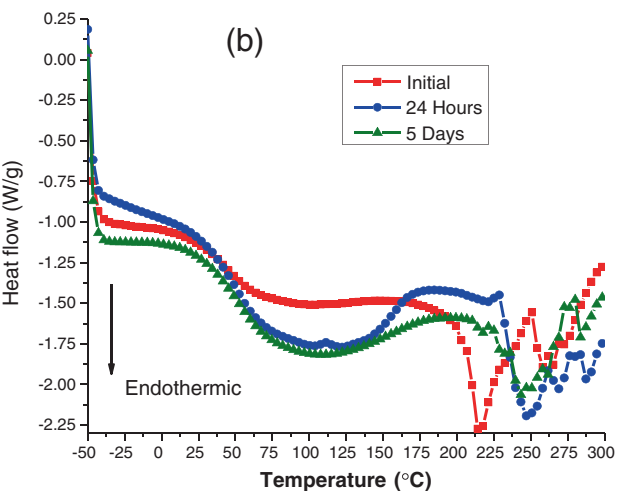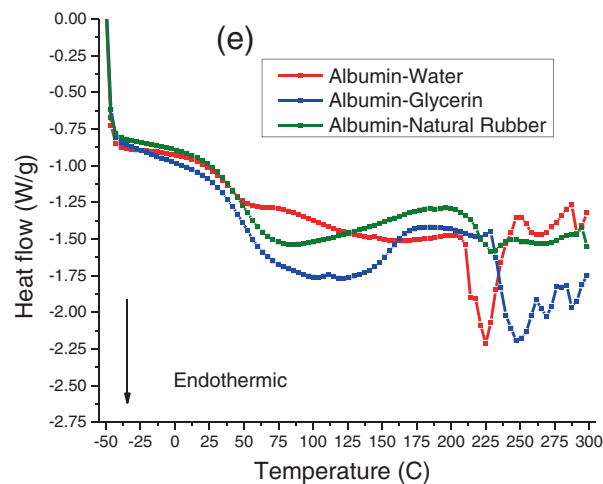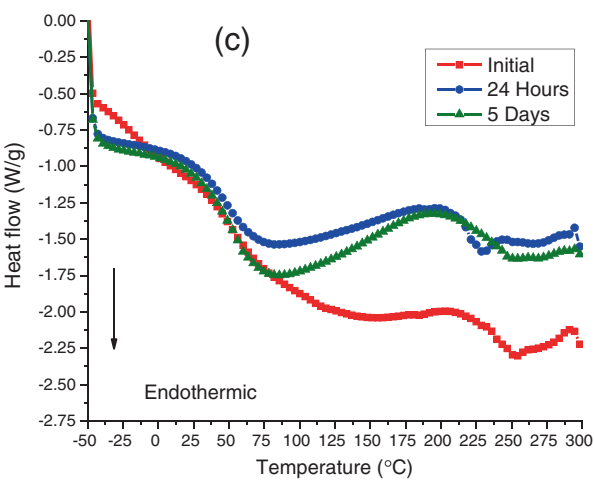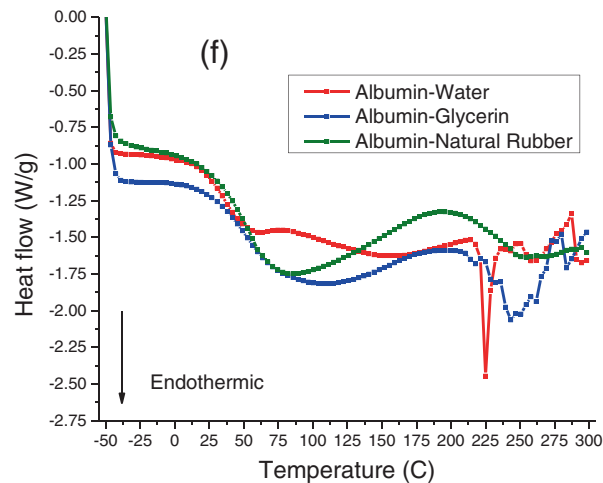

Supplement: Supplementary file 5 — Authors’ original file for figure 5 [file 40204_2013_16_MOESM5_ESM.pdf]

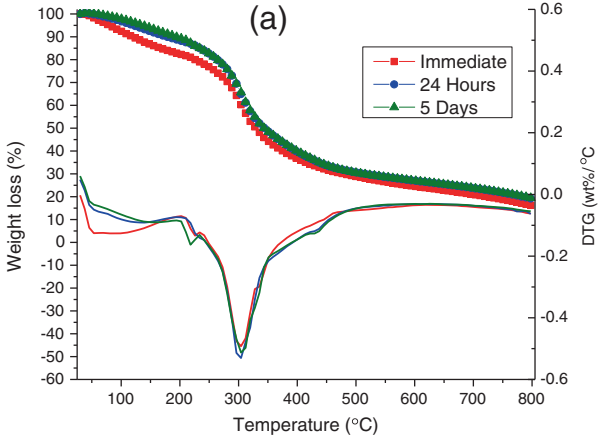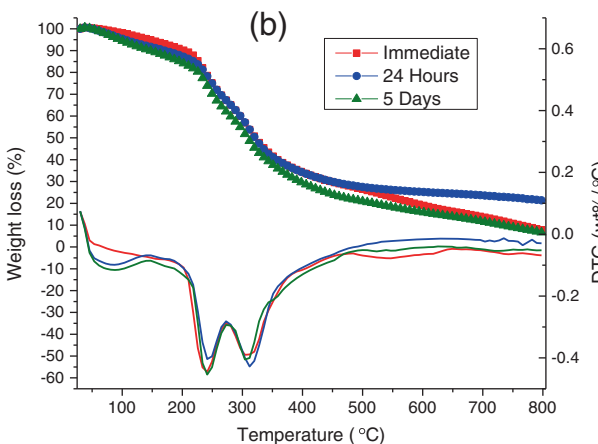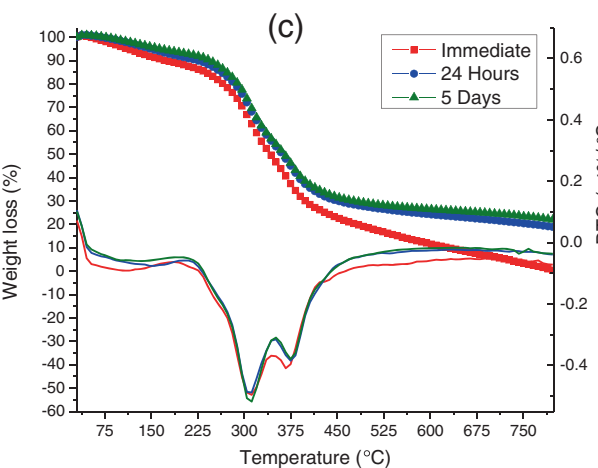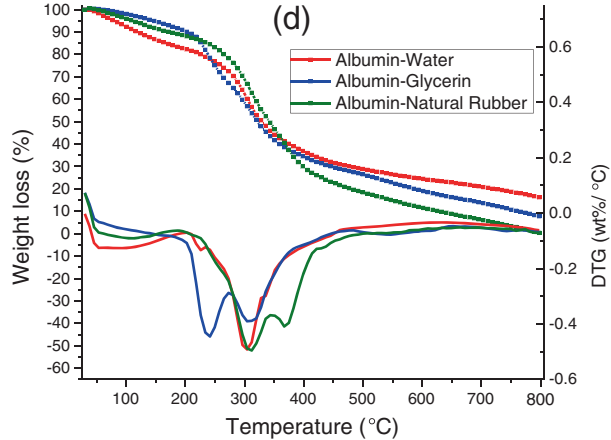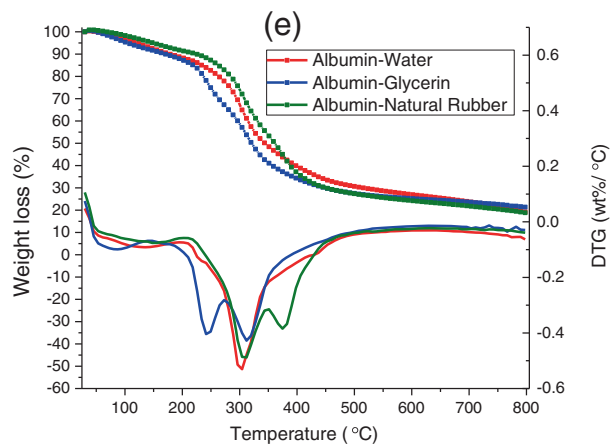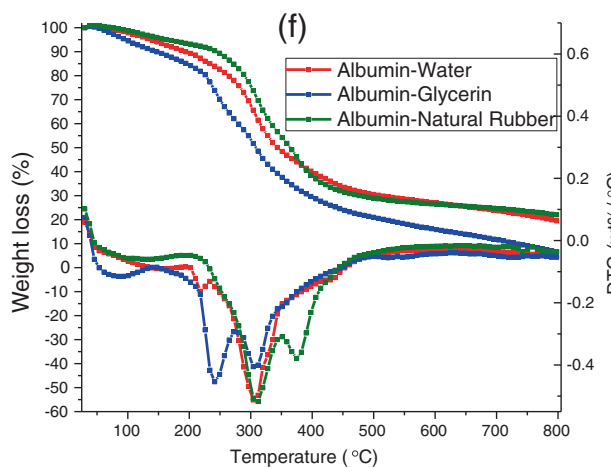

Supplement: Supplementary file 6 — Authors’ original file for figure 6 [file 40204_2013_16_MOESM6_ESM.pdf]

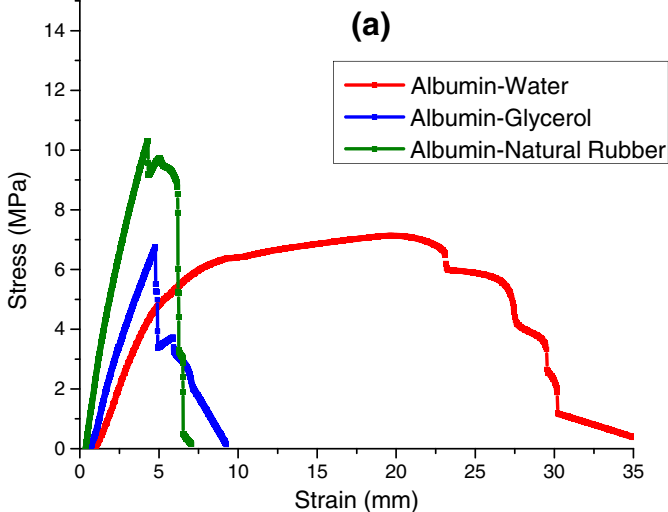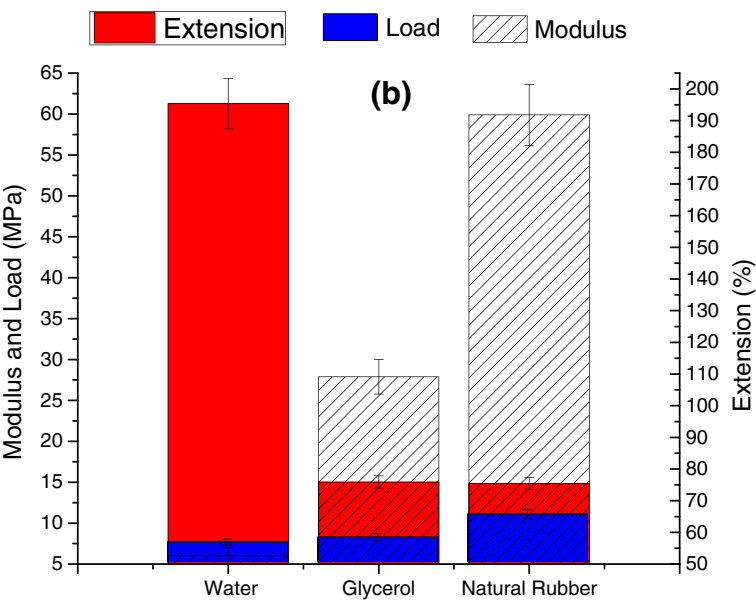

Supplement: Supplementary file 7 — Authors’ original file for figure 7 [file 40204_2013_16_MOESM7_ESM.pdf]

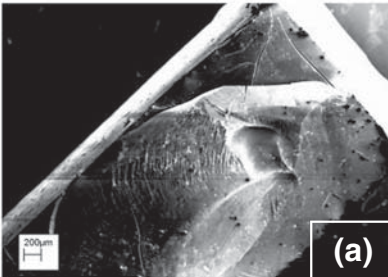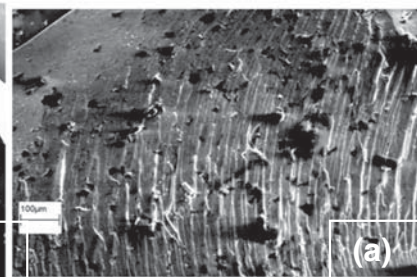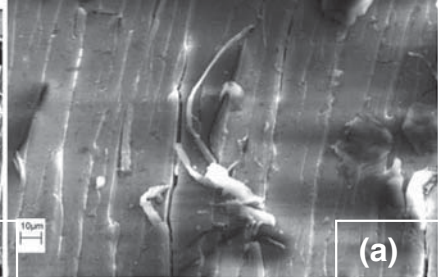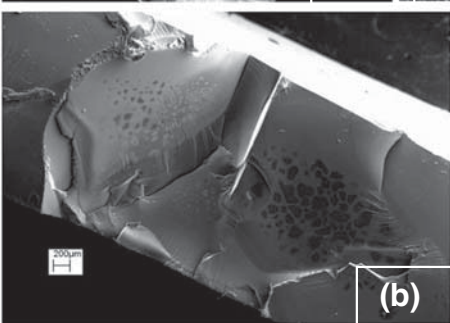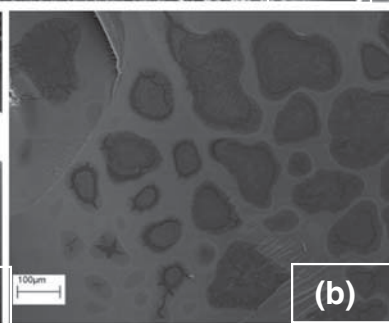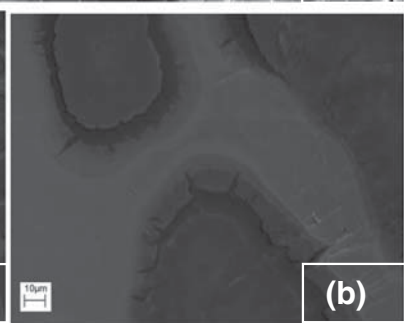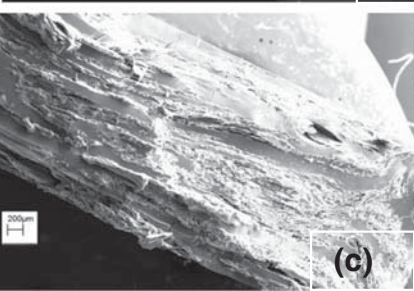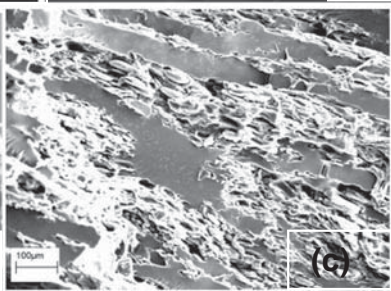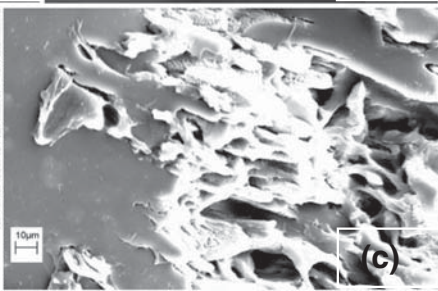

Supplement: Supplementary file 8 — Authors’ original file for figure 8 [file 40204_2013_16_MOESM8_ESM.pdf]
